# Localized bullous pemphigoid in the setting of chronic lymphedema

**DOI:** 10.1016/j.jdcr.2025.11.041

**Published:** 2025-12-05

**Authors:** Mara B. O’Connor, Sophia G. Allison, Nicole Trupiano, Rithu P. Srikantha

**Affiliations:** a Department of Dermatology, Northwestern University, Chicago, Illinois; b Department of Medicine, Division of Dermatology, Albert Einstein College of Medicine, Bronx, New York

**Keywords:** bullous pemphigoid, bullous pemphigoid recurrence, chronic lymphedema, lower extremity edema

## Case

**Fig 1:**
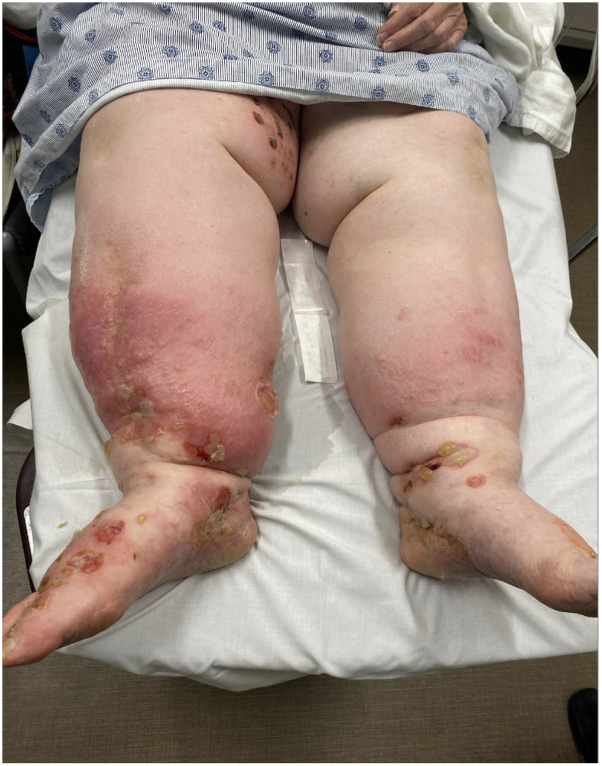
Bilateral lower extremities with tense bullae and background erythema.

80-year-old women with a history of chronic bilateral lymphedema were admitted to the hospital for a 6-month history of widespread blistering. She had tense bullae with surrounding erythema on her bilateral lower extremities and bilateral forearms (Fig 1). A lesional biopsy of the right forearm was performed and demonstrated subepidermal bullae with numerous eosinophils within the blister and dense eosinophilic infiltrate of the dermis with marked edema. Direct immunofluorescence confirmed linear deposits of immunoglobulin (Ig) G1, IgG4, and C3 at the dermal-epidermal junction and she was diagnosed with generalized bullous pemphigoid (BP). Her BP was managed with clobetasol ointment and daily azathioprine. The azathioprine was tapered off over 18 months given improvement in disease with complete remission in 2023.

**Fig 2:**
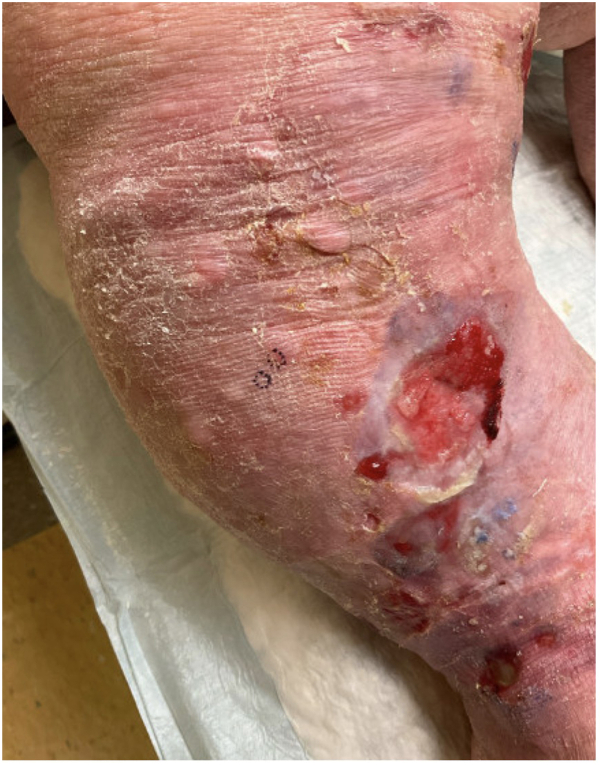
Right lower extremity.

Twelve months after discontinuing azathioprine, the patient presented to clinic with new bullae and worsening edema of the bilateral lower extremities (Fig 2). The bullae were strictly localized to areas affected by lymphedema and clinically appeared consistent with an acute exacerbation of her underlying edema. Given the persistence of her lower extremity symptoms despite continued lymphedema therapy, a perilesional biopsy was performed. Biopsy demonstrated perivascular dermatitis with eosinophils. Direct immunofluorescence demonstrated linear deposits of IgG1, IgG4, and C3 at the dermal-epidermal junction. Furthermore, an immunobullous antibody panel was performed which demonstrated elevated BP180 and 230 IgG antibodies at 66 U/mL and 70 U/mL, respectively.

**What is the most likely diagnosis?**

**A.** Pemphigus vulgaris
**B.** Bullae secondary to lymphedema
**C.** Epidermolysis bullosa simplex
**D.** Localized recurrence of BP
**E.** Porphyria cutanea tarda

## Discussion

The pathology and laboratory findings are consistent with a diagnosis of a localized recurrence of BP which aligned with progression of her lymphedema. She ultimately improved after initiating dupilumab and a short hospitalization for intravenous diuresis. Localized BP is a subset of disease that shares the same histologic and immunologic findings as the generalized form.^1^ Local inflammation and trauma including surgery, radiotherapy, venous stasis, infection, and lymphedema and medications have been reported as triggers for local BP in the literature.^1^^,^^2^^,^^3^^,^^4^ Compared to radiotherapy, which is a well-known trigger of BP with more than 50 cases reported, there are only 4 cases reported with lymphedema as the trigger for BP.^1^^,^^2^^,^^3^^,^^4^ Decreased circulation in the lymphatic system, increased vessel permeability, and cleavage of the dermal-epidermal junction due to hydrostatic pressure are all mechanisms by which lymphedema has been thought to trigger BP.

There is evidence that pemphigoid disease can demonstrate an isomorphic phenomenon akin to the Koebner phenomenon, which may align with the localized recurrence of our patient’s disease only in the areas affected by lymphedema.^5^ The Koebner phenomenon describes the appearance of new skin lesions on previously unaffected skin at sites of trauma that match a patient’s previously established dermatologic disease.^5^ Several case reports propose that the mechanism of the Koebner phenomenon in pemphigoid disease is related to traumatized epithelium exposing new epitopes that may initiate antibody formation or allow already circulating antibodies to attack.^5^ The Koebner phenomenon is primarily associated with acute trauma, but we postulate this may occur secondary to a more acute exacerbation of a chronic inflammatory process like lymphedema.

## Conflicts of interest

None disclosed.

## References

[1] Binitha M.P., Vishnu V.V., Sreekanth S., Reena Mariyath O.K. (2014). Localized bullous pemphigoid on sites of radiotherapy and lymphedema in the same patient. Indian Dermatol Online J.

[2] Janßen S., Homey B., Jansen T.M. (2022). Bullöses Pemphigoid mit Aussparung eines Armes nach axillärer Lymphadenektomie [Bullous pemphigoid with unilateral sparing after axillary lymphadenectomy]. Dermatologie (Heidelb).

[3] Callens A., Vaillant L., Machet M.C. (1993). Localized atypical pemphigoid on lymphoedema following radiotherapy. Acta Derm Venereol.

[4] Perez A., Clements S.E., Benton E. (2009). Localized bullous pemphigoid in a patient with primary lymphoedema tarda. Clin Exp Dermatol.

[5] Rotunda A.M., Bhupathy A.R., Dye R., Soriano T.T. (2005). Pemphigus foliaceus masquerading as postoperative wound infection: report of a case and review of the Koebner and related phenomenon following surgical procedures. Dermatol Surg.

